# Comparative Evaluation of Mechanical Properties and Microleakage of Cention N and Titanium Dioxide Nanoparticles-Enriched Cention N: An In Vitro Study

**DOI:** 10.7759/cureus.51209

**Published:** 2023-12-28

**Authors:** Nancy Irudayaraj, Sinduja Rajamani, Padmapriya Mahalingam, Janani Karunakaran, Afridh Hameedha M, Darcus Evangelin Chandran

**Affiliations:** 1 Department of Conservative Dentistry and Endodontics, Greater Chennai Corporation, Chennai, IND; 2 Department of Conservative Dentistry and Endodontics, Chettinad Dental College and Research Institute, Chennai, IND; 3 Department of Conservative Dentistry and Endodontics, Tamil Nadu Government Dental College and Hospital, Chennai, IND

**Keywords:** tio2-infused cention n, microhardness, microleakage, mechanical properties, cention n

## Abstract

Aim

This study aimed to assess and compare the mechanical properties, including compressive strength, diametral tensile strength, flexural strength, fracture resistance, microhardness, and microleakage, between Cention N (Ivoclar Vivadent, Gurugram, India) and Cention N enriched with titanium dioxide (TiO_2_) nanoparticles.

Methodology

A total of 120 samples were involved in the study, which were split into two experimental groups. For evaluation of each mechanical property, 20 samples were included, of which 10 samples were used for evaluating the properties of Cention N and 10 samples were used for TiO_2 _nanoparticles-enriched Cention N. Samples, formed using Teflon molds, were filled with Cention N and TiO_2_-enriched Cention N powders mixed per the manufacturer's instructions. The universal testing machine (UTM) was used to assess compressive, flexural, diametral tensile strength, and fracture resistance. Microhardness was evaluated using a diamond indenter, while microleakage was examined utilizing a stereomicroscope.

Results

The nanotitania-enriched Cention N showed significantly increased mechanical properties and increased microhardness with the least microleakage.

Conclusion

The inclusion of TiO_2_ in Cention N has proved to yield promising results.

## Introduction

Dental restorative materials are crucial in replacing lost tooth structures. The biological, functional, and aesthetic characteristics of a healthy tooth structure are restored. For more than a hundred years, silver amalgam has been prized in dental practice for its exceptional properties, making it a preferred choice for dental restorations; nonetheless, the amalgam releases vapors that are harmful to humans. Thus, the usage of silver amalgam remains controversial. Hence, new restorative materials have been developed to have similar mechanical properties to silver amalgam but with improved aesthetic properties as well as reduced toxicity [1].

Cention N (Ivoclar Vivadent, Gurugram, India) is a recently developed filling material for direct restoration that is self-curing, resin-based, and tooth-colored. It is an alkaline restorative substance that releases ions that neutralize acids and uses an alkaline filler [2].

Cention N comes in both liquid and powder form and also as capsules. Filler particles and additional initiator components make up the powder. These consist of calcium barium aluminum fluorosilicate glass, calcium fluorosilicate glass, ytterbium trifluoride, and isofiller. The unique isofiller (Tetric N-Ceram technology) in the powder lessens microleakage and shrinkage during polymerization by acting as a shrinkage stress reducer [2].

There are four distinct dimethacrylate monomers and initiators in the liquid. Urethane dimethacrylate (UDMA), tetramethyl-xylylendiurethane dimethacrylate (aromatic aliphatic UDMA), tricyclodecane dimethanol dimethacrylate (DCP), and polyethylene glycol 400 dimethacrylate (PEG-400 DMA) are a few of them [2].

Cention N promises better aesthetics with a certain degree of translucency. The presence of ytterbium fluoride filler endows it with radiopacity. The aesthetic quality of dental restorations is significantly affected by surface roughness. Impressively, Cention N demonstrates higher resistance to surface roughness post-chewing simulation, resulting in enhanced aesthetics [3]. Additionally, thermocycling has been observed to enhance color stability, further highlighting its suitability for prolonged use [4].

While amalgam primarily depends on mechanical retention for stability, Cention N offers versatility in its application. When used without adhesives, it necessitates a retentive preparation akin to that required for amalgam restorations. However, if applied with an adhesive, a different approach emerges, allowing for minimal preparation that aims to preserve maximum natural tooth structure. This method involves etching with phosphoric acid to facilitate bonding and enhance adhesion, emphasizing a more conservative approach to tooth preparation [3]. Adding Cention N's exceptional characteristics, such as its notable compressive and flexural strengths, improved bond strength, and heightened microhardness, make it an excellent and commendable alternative to dental amalgam [2,5-7]. It is inexpensive, simple to use, and possesses ion release properties similar to glass ionomer cement.

Currently, dental research is increasingly focused on the application of nanoparticles. The mechanical characteristics and antibacterial action of the nanoparticles are enhanced when used in combination. An inorganic additive that claims the aforementioned qualities is titanium dioxide (TiO_2_). TiO_2_ nanoparticles are biocompatible and chemically stable. Their potent antimicrobial properties stem from the generation of powerful oxidizing free radicals, such as hydroxyl and superoxide anion radicals. This attribute has shown significant efficacy in reducing the growth of diverse microorganisms, such as *Escherichia coli* and *Staphylococcus aureus* [8]. TiO_2_ has a pro-inflammatory effect through interleukin-1β, which also causes the release of prostaglandin E2, cyclooxygenases 1 and 2, and hepatocyte growth factor (HGF) cells to create significant metabolic alterations in the culture medium [9]. As a result, it is anticipated that Cention N enhanced by nanoparticle TiO_2_ will have better mechanical qualities than Cention N [10].

Contrasting the characteristics between Cention N and Cention N enriched with TiO_2 _nanoparticles can assist the clinician in selecting the most suitable material for the restoration. The objective of this research is to assess and contrast the mechanical characteristics of Cention N and TiO_2_ nanoparticles-enriched Cention N. The null hypothesis was that Cention N enriched with TiO_2_ would neither enhance the mechanical properties nor limit microleakage when used as a restorative material.

## Materials and methods

A total of 120 samples were involved in the study and were split into two experimental groups: Group 1 - Cention N (n = 60) and Group 2 - TiO_2 _nanoparticles-enriched Cention N (n = 60).

For the evaluation of each property, 20 samples were included, of which 10 samples were used for evaluating the properties of Cention N and 10 samples were used for TiO_2_ nanoparticles-enriched Cention N. To assess each property, distinct-sized samples were created using Teflon molds. Unblended Cention N powder was used for Group 1. For the subsequent group, powders were produced by blending Cention N (Ivoclar Vivadent) with TiO_2 _nanoparticles (Matrix Nano, Greater Noida, India) in a proportion of 5% (w/w). The powder and liquid of each group were proportioned and mixed according to the manufacturer’s instructions. These mixtures were poured and then condensed into the molds, covered with two matrix strips and glass slides to prevent air pockets, and left to solidify. Once set, the samples were removed from the molds, excess material was eliminated by grinding with wet 600-grit silicon carbide (SiC) abrasive paper, and the specimens were stored in distilled water before testing. The outcome assessor was blinded about the group allocation.

Compressive strength

**Figure 1:**
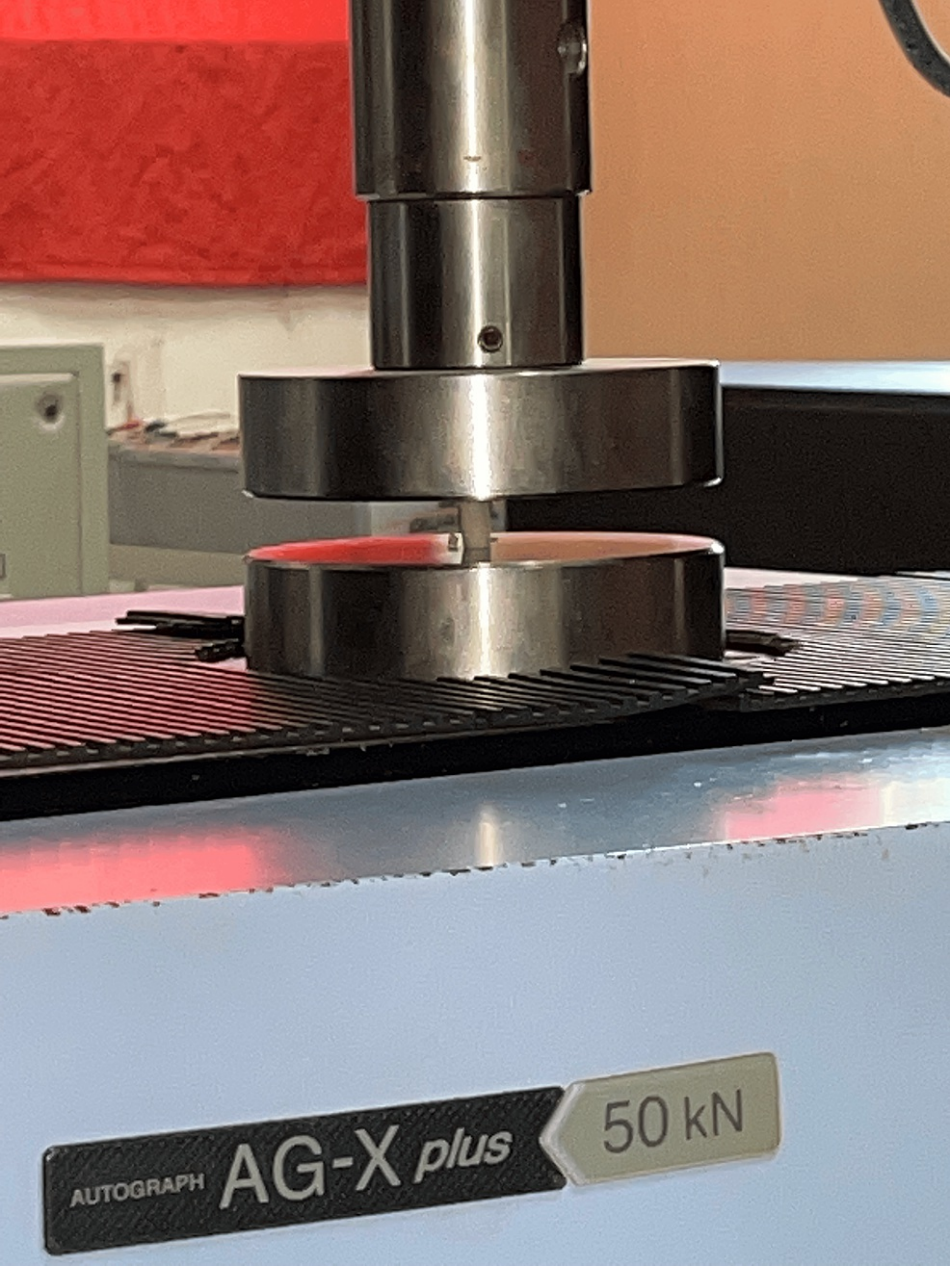
Compressive strength testing

Twenty customized cylindrical samples measuring 12 mm in height and 6 mm in diameter were created and distributed equally, with 10 samples allocated to each of Group 1 and Group 2. These cylinders were tested utilizing a universal testing machine (UTM). The UTM was equipped with a load-measuring cell that monitored the applied load on the samples. The testing proceeded at a controlled crosshead speed of 0.75 ± 0.25 mm per minute until the samples fractured (Figure 1).

Flexural strength

**Figure 2:**
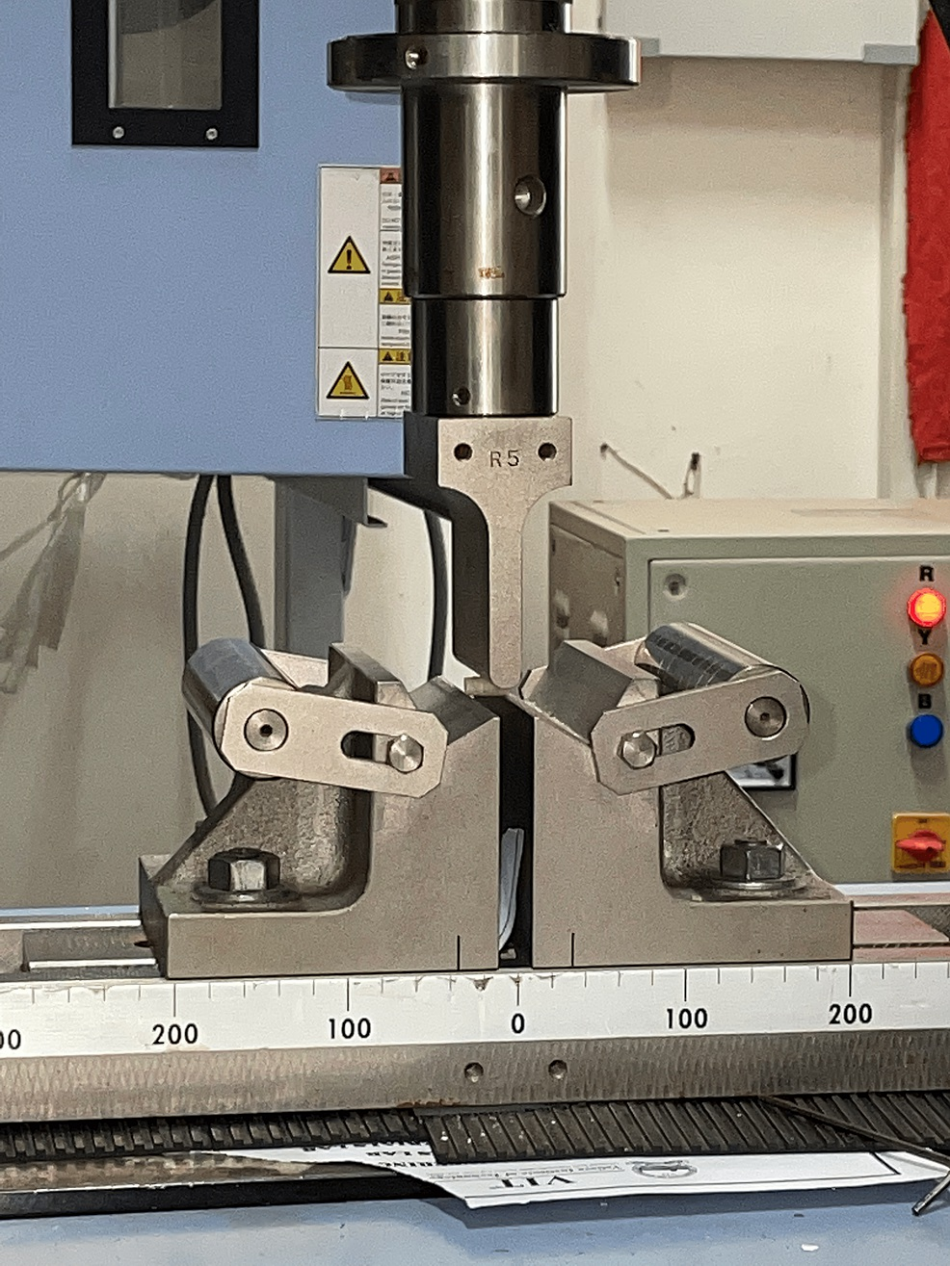
Flexural strength testing

Twenty customized cylindrical samples measuring 12 mm in height and 6 mm in diameter were created and distributed equally, with 10 samples allocated to each of Group 1 and Group 2. These cylinders underwent three-point bending tests utilizing a UTM. The UTM was equipped with a load-measuring cell that monitored the applied load on the samples. The testing proceeded at a controlled crosshead speed of 0.75 ± 0.25 mm per minute until the samples fractured (Figure 2).

Diametral tensile strength

**Figure 3:**
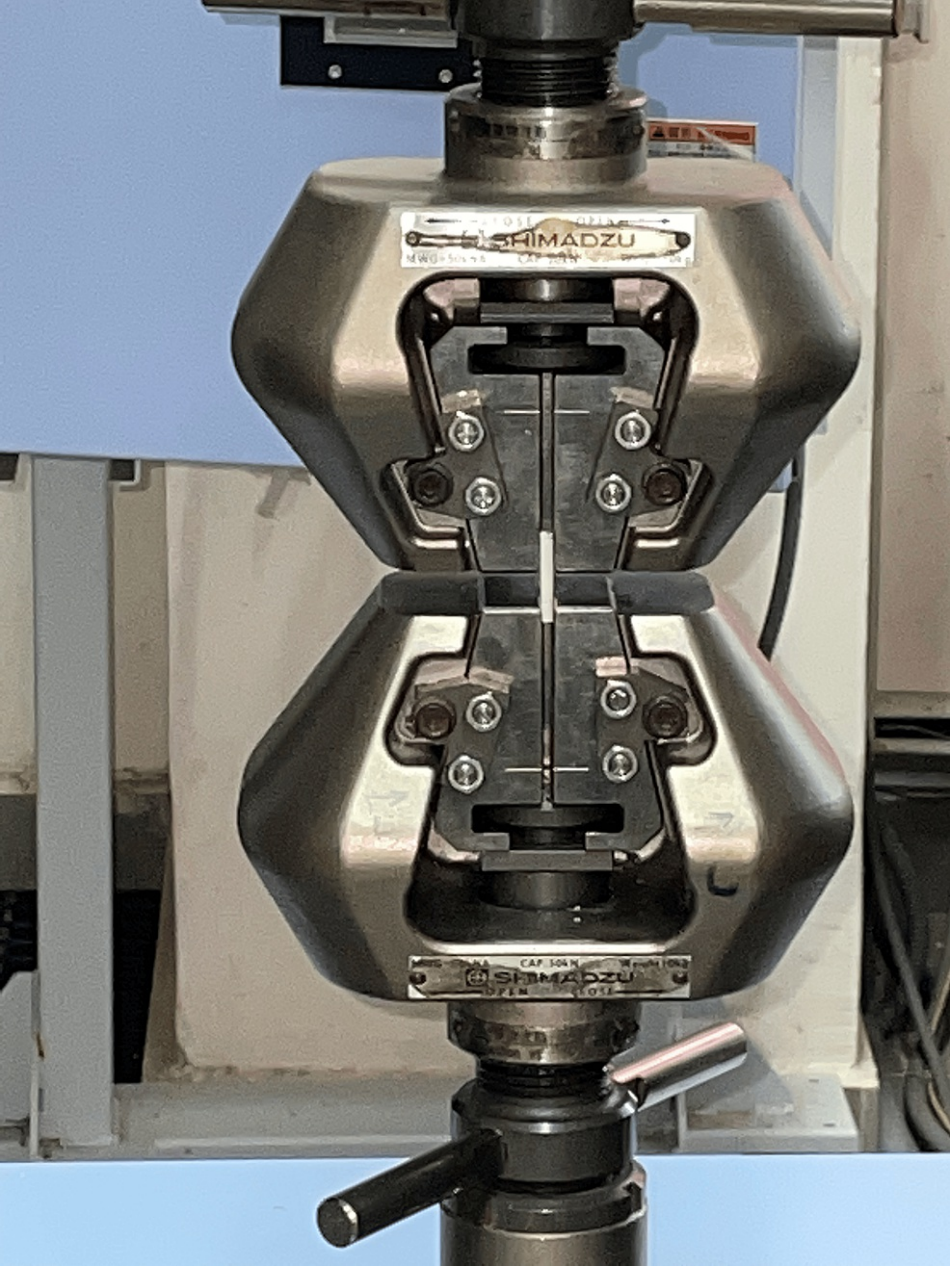
Diametral tensile strength testing

Twenty customized disk-shaped samples measuring 6 mm in diameter and 3 mm in height were manufactured, with 10 samples allocated to both Group 1 and Group 2. These samples were positioned on an Instron UTM (Norwood, MA) with their diameter aligned with the direction of the force. Force, applied at a crosshead speed of 1 mm per minute, was exerted on the pellets until they fractured (Figure 3).

Fracture resistance

**Figure 4:**
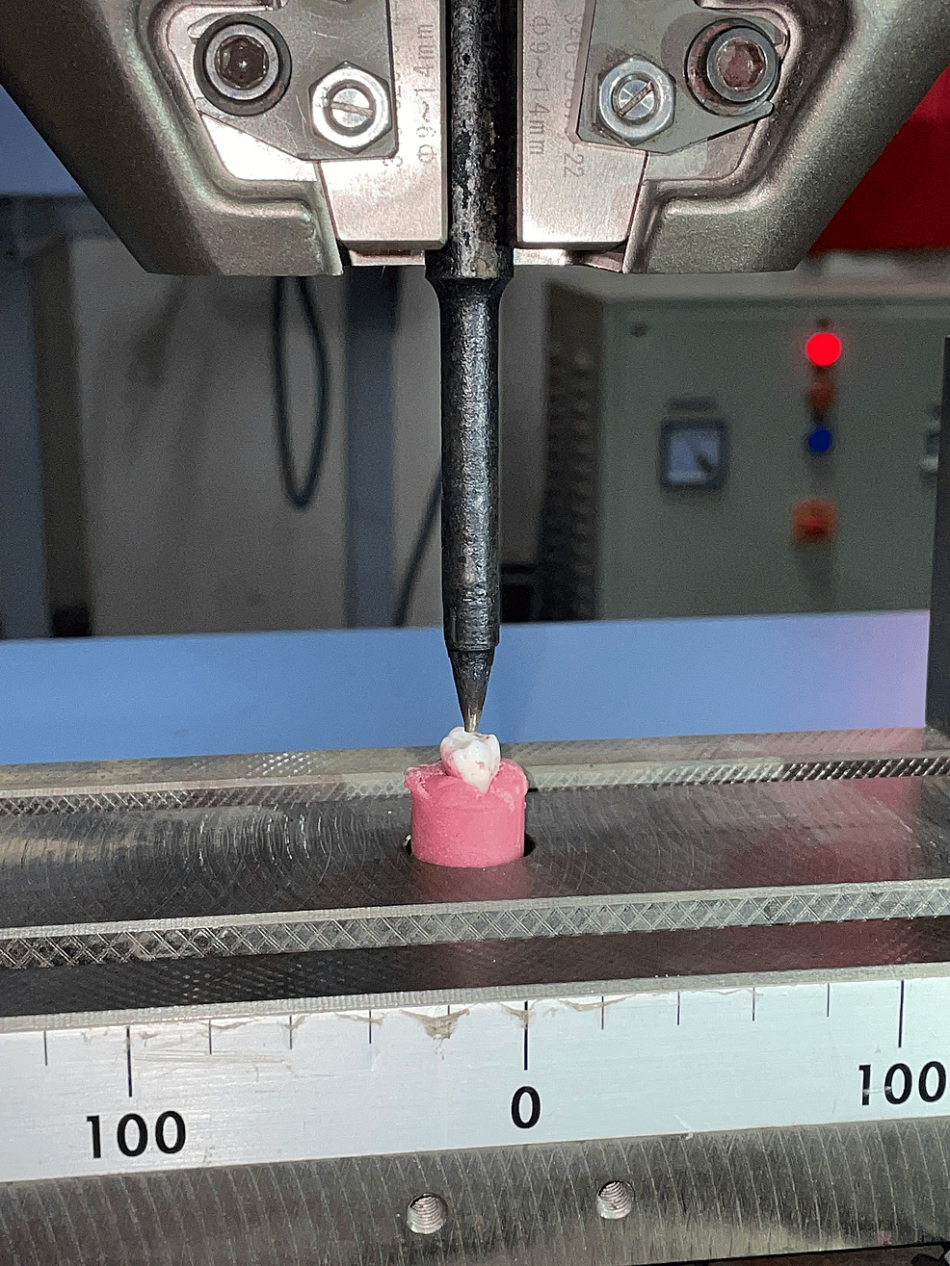
Testing for fracture resistance

A cylindrical mold was utilized to secure each tooth with self-cured acrylic resin, placing them up to 2.0 mm below the cementoenamel junction. Subsequently, the samples underwent compressive loading at a crosshead speed of 1 mm per minute using Torsee's Electronic System UTM. A metal zig placed at the tooth's center facilitated the application of compressive force. The recorded force required to induce fracture was documented (Figure 4).

Vickers microhardness test

**Figure 5:**
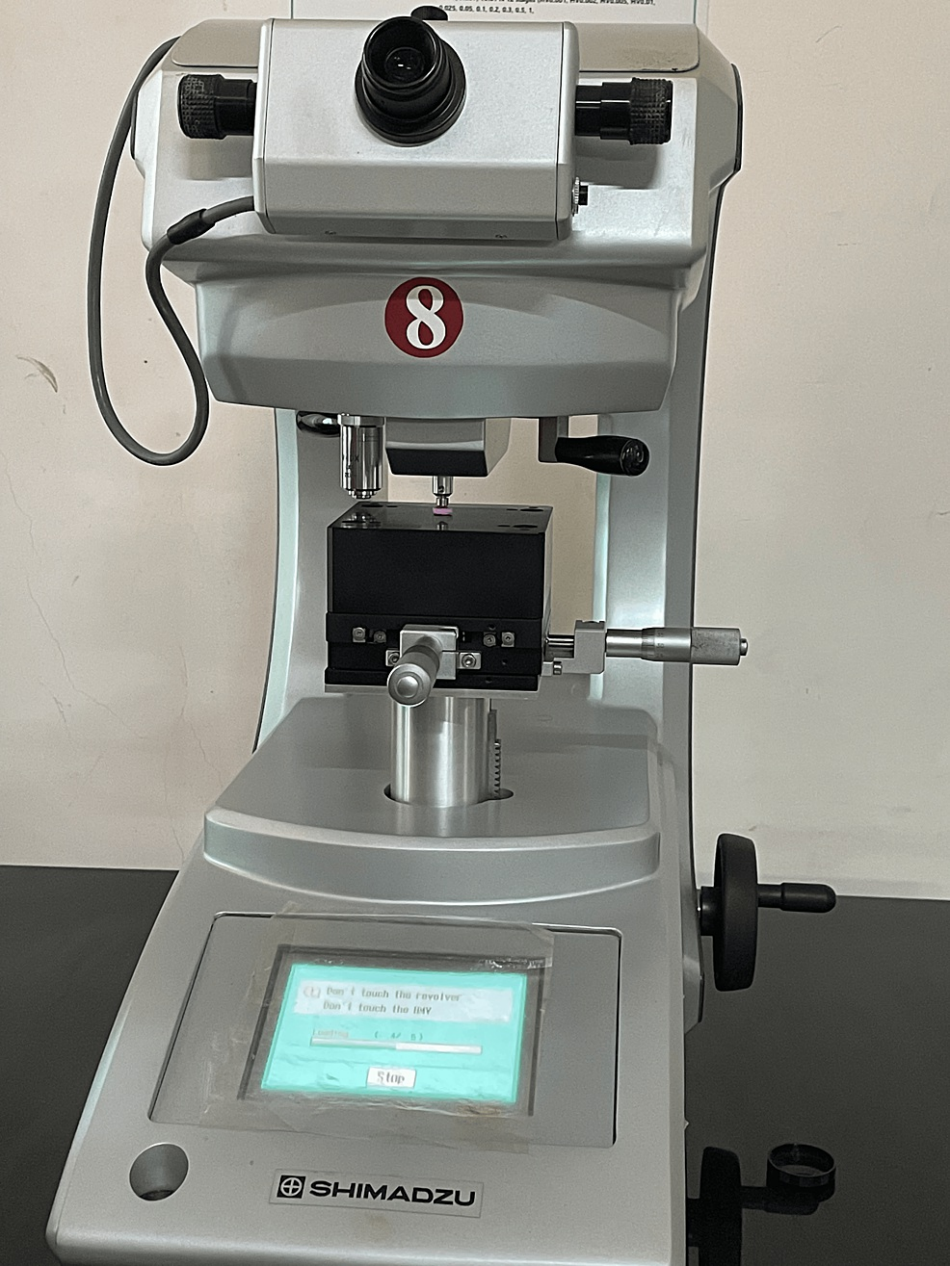
Testing for Vickers microhardness (HMV Microhardness Tester, Shimadzu, Japan)

Twenty customized cylinders measuring 5 mm in diameter and 2 mm in height were created, with 10 samples allocated to both Group 1 and Group 2. These cylinders underwent testing using an ISO 9001:2008 certified diamond indenter in a microhardness tester (HMV Microhardness Tester, Shimadzu, Japan) with a 10 N force. A dwell time of 10 seconds was employed for conducting 10 indentations across the specimens of each group, resulting in a total of 50 indentations for each group (Figure 5).

Microleakage

Twenty human maxillary and mandibular premolars, extracted without any signs of cracks, caries, restorations, or white spot lesions, were carefully chosen. These teeth underwent cleaning and were preserved in distilled water until they were ready for use. Class I cavities were prepared on the occlusal surface of the premolars. The tooth preparations were randomly divided into two groups, each comprising 10 samples. The restoration process involved using specific materials: Group 1 utilized Cention N, while Group 2 employed Cention N enriched with TiO_2_.

After the restorative procedure, all treated teeth were stored in distilled water at 37°C for 24 hours. Subsequently, they were exposed to 200 thermocycles involving temperature variations between 5°C and 55°C for 30-second intervals to replicate oral thermal conditions. Sticky wax was used for sealing to prevent dye leakage, followed by applying two coats of clear nail varnish to cover all crown and root surfaces, leaving 1 mm around the restoration margins.

The samples were then immersed in a 0.5% basic fuchsine dye at 37°C for 24 hours. After washing and drying, each tooth was longitudinally sectioned through the center of the restoration using a diamond disk under water coolant. All sections were observed under a stereomicroscope (Labomed Luxeo 2S Stereomicroscope, Los Angeles, CA) at 20× magnification. The section exhibiting the most significant microleakage was chosen for evaluation. The distance of leakage from the margins to the identified point was measured in millimeters.

Munro, Hilton, and Hermesch's scoring system was used for scoring the leakage in the present study. Score 0: no evidence of microleakage; score 1: penetration of dye up to half of cavity depth; score 2: microleakage more than half of the depth of the cavity wall; score 3: leakage of dye involving axial wall.

**Figure 6:**
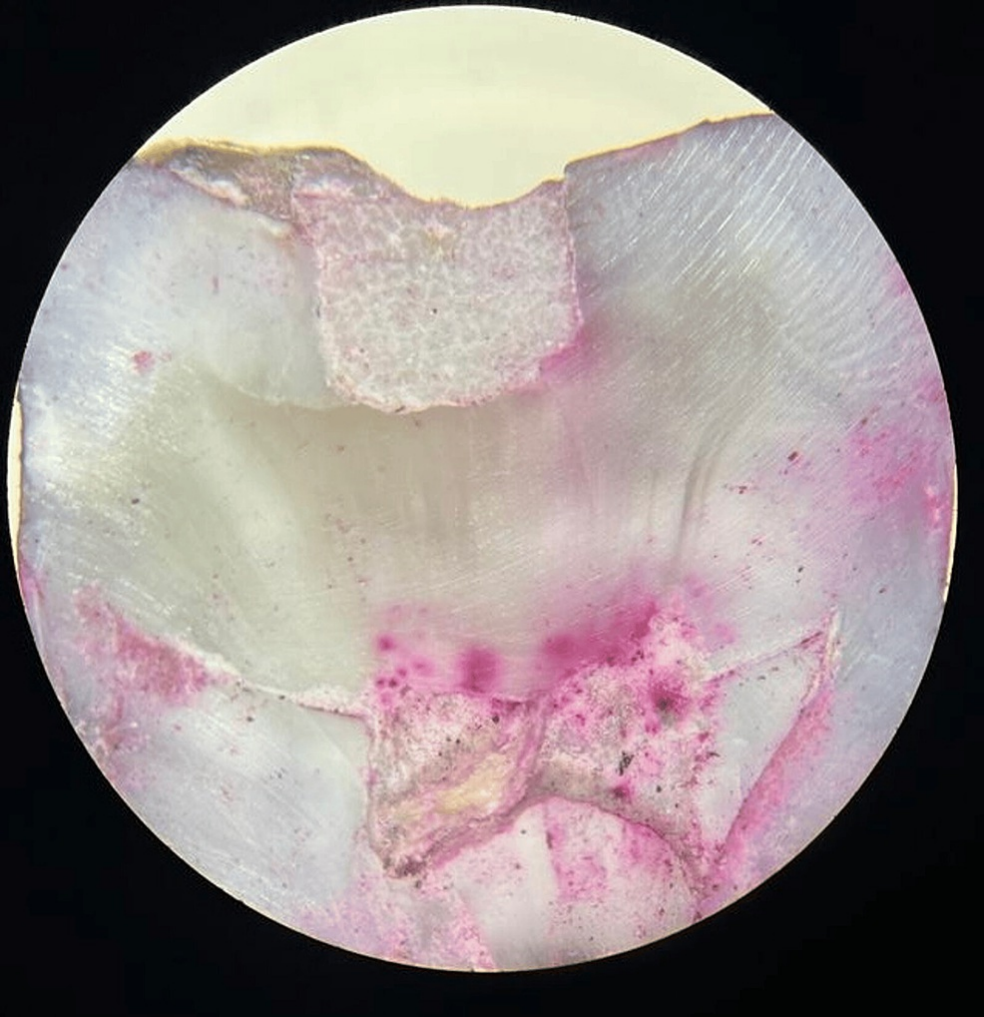
Microleakage evaluation using a stereomicroscope (Labomed Luxeo 2S Stereomicroscope, Los Angeles, CA) at 20x magnification

The microleakage scores for all groups were noted and assessed based on these observations (Figure 6).

Statistical analysis

Mean values and standard deviation of the different mechanical properties of Cention N and nanoparticles-enriched Cention N with TiO2 were estimated using an independent sample t-test. Descriptive statistics of frequency distribution and percentages for microleakage were calculated for both groups of samples restored with Cention N and nanoparticles-enriched Cention N with TiO2 using a chi-square test. A value of p < 0.05 was deemed as statistically significant.

## Results

Compressive strength

Table 1 indicates the mean compressive strength for Cention N (193.05 MPa) and TiO2 nanoparticles-enriched Cention N (241.12 MPa), which is statistically significant (p = 0.0001).

**Table 1:** Mean and standard deviation of compressive strength (MPa) * Significant (p < 0.05).

Material	n	Mean	SD	95% CI for mean		P-value
				Lower bound	Upper bound	
Cention N	10	193.05	15.71	181.81	204.28	0.0001*
Cention N + TiO_2_	10	241.12	13.80	231.25	250.99	

Flexural strength

Table 2 indicates the mean flexural strength for Cention N (91.91 MPa) and TiO2 nanoparticles-enriched Cention N (118.16 MPa), which is statistically significant (p = 0.0001).

**Table 2:** Mean and standard deviation of flexural strength (MPa) * Significant (p < 0.05).

Material	n	Mean	SD	95% CI for mean		P-value
				Lower bound	Upper bound	
Cention N	10	91.91	7.85	86.30	97.53	0.0001*
Cention N + TiO_2_	10	118.16	4.89	114.66	121.65	

Diametral tensile strength

Table 3 indicates the mean diametral tensile strength for Cention N (42.02 MPa) and TiO2 nanoparticles-enriched Cention N (51.28 MPa), which is statistically significant (p = 0.0001).

**Table 3:** Mean and standard deviation of diametral tensile strength (MPa) * Significant (p < 0.05).

Material	n	Mean	SD	95% CI for mean		P-value
				Lower bound	Upper bound	
Cention N	10	42.02	1.08	41.24	42.79	0.0001*
Cention N + TiO_2_	10	51.28	3.28	48.93	53.63	

Fracture resistance

Table 4 indicates the mean fracture resistance for Cention N (871.16 N) and TiO2 nanoparticles-enriched Cention N (1229.95 N), which is statistically significant (p = 0.0001).

**Table 4:** Mean and standard deviation of fracture resistance (N) * Significant (p < 0.05).

Material	n	Mean	SD	95% CI for mean		P-value
				Lower bound	Upper bound	
Cention N	10	871.16	43.01	840.39	901.93	0.0001*
Cention N + TiO_2_	10	1229.95	157.18	1117.51	1342.39	

Vickers microhardness test

Table 5 indicates the mean microhardness for Cention N (66.73 VHN) and TiO2 nanoparticles-enriched Cention N (106.35 VHN), which is statistically significant (p = 0.0001).

**Table 5:** Mean and standard deviation of Vicker's microhardness (VHN) * Significant (p < 0.05).

Material	n	Mean	SD	95% CI for mean		P-value
				Lower bound	Upper bound	
Cention N	10	66.73	11.22	58.70	74.76	0.0001*
Cention N + TiO_2_	10	106.35	16.81	94.32	118.38	

Microleakage

Table 6 indicates that about 70% of Cention N samples showed dye penetration of about 3 mm indicating higher microleakage. On the other hand, about 70% of samples containing TiO2 nanoparticles-enriched Cention N showed no dye penetration indicating no microleakage, which is statistically significant (p = 0.001).

**Table 6:** Microleakage of Cention N and nanoparticles-enriched Cention N * Significant (p < 0.05).

Material	N	Number of samples with no dye penetration		Number of samples with dye penetration unto 1 mm		Number of samples with dye penetration unto 2 mm		Number of samples with dye penetration unto 3 mm		P-value
		N	%	N	%	N	%	N	%	
Cention N	10	0	0	1	10	2	20	7	70	0.001*
Cention N with TiO_2_	10	7	70	3	30	0	0	0	0	

## Discussion

There has been increasing demand for tooth-colored restorative materials due to their aesthetic appeal. Nevertheless, it is crucial to also include the strength of these materials as a factor, when selecting them to ensure they align with the specific clinical requirements. The various mechanical characteristic of a material significantly influences its effective functionality [11,12]. Incomplete sealing at the interface of tooth restorations results in the development of micro gaps, consequently causing microleakage.

Cention N showcases an array of enhanced mechanical properties that distinguish it prominently from other conventional restorative materials. Its superior properties are attributed to a comprehensive polymer network density achieved through the integration of cross-linking methacrylate monomers alongside a robust, effective self-curing initiator. This unique composition ensures a high degree of uniform polymerization throughout the restoration, endowing it with exceptional strength and resilience. Additionally, the incorporation of specialized fillers, like isofiller, functions as a stress-relieving component, effectively mitigating shrinkage forces that commonly afflict other materials. This reduction in volumetric shrinkage substantially minimizes the potential for microleakage, further solidifying Cention's reputation for not only superior mechanical integrity but also heightened longevity and reliability in dental restorations [13,14].

Nanoparticles in dentistry have garnered recent attention owing to their antibacterial, antiviral, and anti-inflammatory traits. Within this context, employing safer nanoparticles with fewer gaps, thereby enhancing resistance, could offer more substantial advantages [15-18]. Among metallic nanoparticles, the inclusion of TiO_2_ has demonstrated robust antibacterial properties, reinforcing its physicomechanical characteristics [19-22].

The sealing and bonding property of TiO_2_ in restorative materials plays a role in reducing microleakage at the restoration interface. TiO_2_ nanoparticles potentially decrease polymerization shrinkage by minimizing the shrinkage stress. This is attributed to the high surface area-to-volume ratio of TiO_2_ nanoparticles. When dispersed within the matrix, this larger surface area provides more interaction sites between the nanoparticles and the surrounding matrix, resulting in improved stress distribution and aids in enhancing the physical and mechanical characteristics of the material, thereby fostering an improved seal in the midst of tooth and the restorative material [23-30].

In this study, upon comparing multiple mechanical properties such as compressive strength, flexural strength, diametral tensile strength, fracture resistance, and microhardness between two groups, we observed that Cention N enriched with TiO_2_ nanoparticles displayed notably higher values. This suggests improved mechanical properties for Cention N when supplemented with TiO_2_ nanoparticles. The enhanced mechanical characteristics observed could be credited to how TiO_2_ particles disperse within the Cention N matrix, effectively filling voids and fostering a more consistent structure. This optimized packing density plays a pivotal role in bolstering the material's overall strength. Moreover, this uniform distribution effectively impedes dislocation movement, leading to an improvement in the material's hardness.

Similarly, in the comparison of microleakage between the two groups, findings revealed that among samples restored with Cention N, 70% exhibited dye penetration up to 3 mm, 20% displayed dye penetration up to 2 mm, and approximately 10% showed dye penetration up to 1 mm. Conversely, among samples restored with Cention N enriched with TiO_2_, 70% showed no dye penetration, while 30% displayed dye penetration up to 1 mm. These outcomes indicate that Cention N enriched with TiO_2_ exhibited significantly reduced or no dye penetration, suggesting minimal to no microleakage. The reduction in microleakage could be linked to the enhanced adherence between the dental cement and the tooth surface. This strengthened bonding establishes a more secure seal, effectively blocking the ingress of microorganisms and fluids between the restoration and the tooth, consequently minimizing the occurrence of microleakage. It was initially hypothesized that TiO2 nanoparticles-enriched Cention N would neither enhance the mechanical properties nor limit microleakage. However, the significant results indicate that TiO_2_ nanoparticles-enriched Cention N not only enhanced the mechanical properties but also limited microleakage. Hence the null hypothesis has been rejected.

The current study's constraint lies in its in vitro nature, as the functionality and outcomes of the restorative material can diverge when applied in oral environments. In an in vitro setting, replicating oral conditions proves challenging, leading to variations in the material's performance compared to oral situations. Moreover, the evaluation of physical properties was conducted on a limited number of materials, and also a single sample was not subjected to all the tests. Thus, additional extensive clinical studies involving larger sample sizes are imperative to provide a more comprehensive insight into the material's behavior in actual circumstances.

## Conclusions

Considering the confines of this analysis, it appears that the TiO_2_-enriched nanoparticles hold significant promise. The addition of TiO_2_ nanoparticles appears to yield enhanced compressive strength, flexural strength, diametral tensile strength, fracture resistance, and microhardness while also maintaining minimal microleakage. Moreover, a more extensive examination of material interactions affecting the behavior of Cention N, alongside investigations into its biocompatibility and long-term stability subsequent to the inclusion of nano titania, ought to be pursued.
